# Temporal Dynamics of Gene Expression During Endothelial Cell Differentiation From Human iPS Cells: A Comparison Study of Signalling Factors and Small Molecules

**DOI:** 10.3389/fcvm.2018.00016

**Published:** 2018-03-14

**Authors:** Heini Belt, Jonna K. Koponen, Tuija Kekarainen, Katja A. Puttonen, Petri I. Mäkinen, Henri Niskanen, Joni Oja, Galina Wirth, Jari Koistinaho, Minna U. Kaikkonen, Seppo Ylä-Herttuala

**Affiliations:** ^1^A. I. Virtanen Institute for Molecular Sciences, University of Eastern Finland, Kuopio, Finland; ^2^Kuopio Center for Gene and Cell Therapy, Kuopio, Finland; ^3^FinVector Vision Therapies Oy, Kuopio, Finland; ^4^Heart Center and Gene Therapy Unit, Kuopio University Hospital, Kuopio, Finland

**Keywords:** endothelial differentiation, cell therapy, iPS cells, RNA sequencing, transcription factors, cardiovascular diseases

## Abstract

Endothelial cell (EC) therapy may promote vascular growth or reendothelization in a variety of disease conditions. However, the production of a cell therapy preparation containing differentiated, dividing cells presenting typical EC phenotype, functional properties and chemokine profile is challenging. We focused on comparative analysis of seven small molecule-mediated differentiation protocols of ECs from human induced pluripotent stem cells. Differentiated cells showed a typical surface antigen pattern of ECs as characterized with flow cytometry analysis, functional properties, such as tube formation and ability to uptake acetylated LDL. Gene expression analysis by RNA sequencing revealed an efficient silencing of pluripotency genes and upregulation of genes related to cellular adhesion during differentiation. In addition, distinct patterns of transcription factor expression were identified during cellular reprogramming providing targets for more effective differentiation protocols in the future. Altogether, our results suggest that the most optimal EC differentiation protocol includes early inhibition of Rho-associated coiled-coil kinase and activation of cyclic AMP signaling, and inhibition of transforming growth factor beta signaling after mesodermal stage. These findings provide the first systematic characterization of the most potent signalling factors and small molecules used to generate ECs from human induced pluripotent stem cells and, consequently, this work improves the existing EC differentiation protocols and opens up new avenues for controlling cell fate for regenerative EC therapy.

## Introduction

Atherosclerotic coronary artery disease (CAD) and peripheral arterial disease (PAD) are leading causes of morbidity and mortality worldwide (1). Adjuvant regenerative therapies to promote therapeutic angiogenesis are needed because current interventions are insufficient in patients with severe disease (2–4). It is also important to promote re-endothelialization and prevent late-stent thrombosis related to conventional therapies (4). In addition, ischemic stroke patients would benefit from efficient neovascularisation after ischemic cerebral injury (5). Therapeutic angiogenesis can be achieved with gene therapy (6) or cell therapy (4). Cell therapy could provide an effective means to enhance therapeutic angiogenesis to restore blood flow in ischemic areas (7, 8).

Endothelial cells (EC) are metabolically active and have a central role in the homeostatic control of angiogenesis, blood pressure, inflammatory cell recruitment, platelet activation, coagulation pathways and oxidative stress (9–12). Vascular growth, remodeling and maturation involve EC migration, proliferation, differentiation to arterial, venous, lymphatic or other special subtypes of ECs, extracellular matrix (ECM) modifications and recruitment of supportive cells (9, 13). In EC-based therapy, these cells could promote therapeutic angiogenesis by secreting protective, proangiogenic cytokines and growth factors that further modulate vascular function, angiogenesis, vascular regeneration and tissue homeostasis (7–9, 14).

EC therapy was first demonstrated by Asahara and colleagues when they isolated endothelial progenitor cells (EPC) from human peripheral blood (PB) (15). Since then, EC therapy research has expanded to many other cell types including bone barrow mononuclear cells (BMNC) (16), hematopoietic stem cells, mesenchymal stem cells and cardiac progenitor cells (4). In addition, ECs derived from embryonic stem cells (ESC) (17) or induced pluripotent stem cells (iPSC) (18, 19) have been extensively studied (20, 21). In theory, the amount of ECs differentiating from pluripotent stem cells (PSC) is limitless which makes them an attractive source of therapeutic cells for treating ischemic diseases.

Several cell culture protocols aiming to produce therapeutic ECs from PSCs have been published (22–33). Major efforts have been done to efficiently guide stem cells to functional, immune-compatible vascular cells. However, multistep manufacturing processes may be vulnerable and could reduce reproducibility of the cell preparations. The goal regarding future patient treatment is to have a cost-effective and consistent large scale cell culture process to obtain safe and therapeutically active ECs. In spite the numerous studies done in the field of EC therapy, an optimal *in vitro* cell culture method for producing therapeutic ECs still remain elusive (22, 26, 34).

In this work, we systematically tested and compared the effect of the most potent published signalling factors and small molecules used to generate ECs from human iPSC (hiPSC). Tested molecules included factors already known to drive EC differentiation, such as Rho-associated coiled-coil kinase (ROCK) inhibitor (25), transforming growth factor beta (TGFβ) inhibitor (24, 35), cyclic adenosine monophosphate (cAMP) analog 8-Br-cAMP (31) and bone morphogenic protein 4 (BMP-4) (30), which were used in seven different combinations. Successful differentiation to ECs was confirmed by cell morphology, phenotypic analyses and functional assays. RNA sequencing (RNA-Seq) was used to gain insight into the changing transcriptome during the differentiation from hiPSC to ECs. Our analysis demonstrated extensive changes in genes related to focal adhesion and regulation of pluripotency. As a proof of the success of the EC differentiation, major EC-specific transcription factors (TFs) were highly expressed in most differentiation groups. Comparison of mature EC gene expression profiles suggested that the most relevant factors in EC differentiation are the activation of cAMP signalling pathway already in the beginning of differentiation process, and the inhibition of TGFβ signalling after the mesodermal differentiation. The inhibition of ROCK signalling was also crucial as it has been proven to be essential to EC proliferation and differentiation from PSCs (25). In conclusion, this study provides the first comprehensive comparison of the effects of signalling factors and small molecules used in EC differentiation protocols on EC phenotype and transcriptome. The knowledge gained here could help to design more efficient EC production methods for regenerative therapy applications.

## Material and Methods

### HiPSC

Human induced pluripotent stem cell line UEFhfiPSC1.4 (36) was derived using lentiviral transduction of Yamanaka transcription factors Oct4, Klf4, Sox2 and c-Myc (18) into fibroblasts isolated from a skin sample taken during cecarean sectioning of a volunteer mother (36). Generation and testing of the UEFhfiPSC1.4 cell line has been described in detail elsewhere and the cells passed all pluripotency tests and differentiated well into any cell type (36, 37). These hiPSCs were cultured in a serum-free stem cell medium supplemented with 20% KnockOut^™^ Serum Replacement (GIBCO) and 8 ng/ml basic fibroblast growth factor (FGF-2) (R&D Systems) (38) on a feeder cell layer of mitotically inactivated foreskin fibroblasts (ATCC, CRL-2429) (36, 38), or in Essential 8 hESC cell culture media (Life Technologies) on Matrigel™ basement membrane matrix (Corning, growth factor reduced, phenol red free) supplemented with 50 IU/ml penicillin (Invitrogen) and 50 µg/ml streptomycin (Invitrogen). Medium was changed daily or every other day and cells were mechanically passaged once a week in the presence of 10 µg/ml ROCK inhibitor Y-27632 (Sigma-Aldrich) (39), or approximately 1–2 times a week with EDTA solution (Life Technologies, 0.5 M pH 8.0). To avoid feeder cell contamination before EC differentiation, UEFhfiPSC1.4 cell colonies were moved from a feeder cell layer to a feeder-free culture employing Matrigel® hESC-Qualified Matrix coating (BD Biosciences) and mTeSR^™^1 medium (STEMCELL Technologies) supplemented with 50 IU/ml penicillin (Invitrogen) and 50 µg/ml streptomycin (Invitrogen). Culturing of hiPSCs in these conditions was done according to a technical manual of STEMCELL Technologies or Life Technologies (Culturing Pluripotent Stem Cells (PSCs) in Essential 8™ Medium).

### HUVECs as Positive EC Controls

Human umbilical vein endothelial cells (HUVEC) were used as positive EC control in flow cytometric analyses and assays for functional EC characterization. Umbilical cords were collected from volunteermothers according to a protocol approved by Ethics Committee of the Kuopio University Hospital (Kuopio, Finland, license number 341/2015).

HUVECs were isolated from umbilical cord samples as previously described (40). HUVECs were cultured in endothelial cell growth medium (Thermo Scientific) on fibronectin-gelatin coating (10 µg/ml, 0.05%; Sigma-Aldrich).

### Differentiation of hiPSCs to ECs

HiPSC colonies were cut with a scalpel from Matrigel-coated dishes under a stereomicroscope and pieces of hiPSC colonies were washed with phosphate buffered saline (PBS). Cells were dissociated with Accutase (Sigma-Aldrich) and approximately 0.7 × 10^4^ cells per cm^2^ were plated on a fibronectin-gelatin (10 µg/ml, 0.05%; Sigma-Aldrich) coated dish. Cells were cultured in serum-free EBM™-2 Endothelial basal medium-2 (Lonza CC-3156) with EGM^™^-2 SingleQuots medium supplement with human epidermal growth factor (hEGF), recombinant human long R3 insulin like growth factor-*1 (*R3-IGF-1), ascorbic acid, hydrocortisone, human FGF-2, heparin and antibiotics GA-1000 (Lonza CC-4176). From this supplement, fetal bovine serum (FBS) and vascular endothelial growth factor (VEGF) were discarded to obtain serum free and VEGF-controlled cell culture conditions. Knockout serum replacement (GIBCO) was used at the final concentration of 20 µl/ml and recombinant human VEGF-A 165 (R&D Systems) was used at the final concentration of 200 ng/ml. Following small molecules and growth factors were used in different combinations and time courses: 10 µM TGFβ inhibitor SB431542 (Tocris Bioscience), 10 µM ROCK inhibitor Y-27632 (Sigma-Aldrich), 20 ng/ml recombinant human BMP-4 (R&D Systems) and 0.25 mM 8-Br-cAMP (SB 431542, Sigma-Aldrich). Cell culture medium was changhed every second day, and differentiating ECs were passaged every 4–6 days using Accutase (Sigma-Aldrich).

In our cell differentiation protocol, we hypothesize that differentiation stages advance from PSC stage through mesodermal commitment (differentiation day 5) towards mature EC stage (differentiation day 15) (22). Different cell culture conditions are described in Table 1. In all groups without the TGFβ inhibitor at day 1 during the differentiation, it was added at day 4 as TGFβ inhibition has been shown to enhance EC vascular identity after mesodermal fate (24). In those groups with BMP-4 at day 1, it was removed at day 4 since it promotes the mesodermal commitment from PSCs (30).

**Table 1:** EC treatment groups.

**Group name abbreviation**	**ROCK-inhibitor**	**TGFβ-inhibitor**	**8-Br-cAMP**	**BMP-4**
**R**	+	−/added day 4	−	−
**RT**	+	+	−	−
**RB**	+	−/added day 4	−	+/removed day 4
**RC**	+	−/added day 4	+	−
**RTB**	+	+	−	+/removed day 4
**RTC**	+	+	+	−
**RTCB**	+	+	+	+/removed day 4

R = ROCK inhibitor, RT = ROCK inhibitor + TGFb inhibitor, RB = ROCK inhibitor + BMP-4, RC = ROCK inhibitor + 8 Br-cAMP, RTB = ROCK inhibitor + TGFb inhibitor + BMP-4, RTC = ROCK inhibitor + TGFb inhibitor + 8 Br-cAMP and RTCB = ROCK inhibitor + TGFb inhibitor + 8 Br-cAMP + BMP-4.

### Flow Cytometry

For analyses, cells were detached with Accutase (Sigma-Aldrich) and incubated at +37°C for 5 min. Blocking was done using 2% FBS-PBS at +4°C for 20 min followed by fixing in 1% paraformaldehyde (PFA). Following antibodies (BD Biosciences) were used for staining according to manufacturer’s instructions: mouse anti-human CD31-R-phycoerythrin (PE), mouse anti-human CD309-PE, mouse anti-human CD34-PE, mouse anti-human CD144-fluorescein isothiocyanate (FITC) and mouse anti-endothelial nitric oxide synthase (eNOS)-PE. For intracellular staining of eNOS and anti-Von Willebrand Factor (vWF)-FITC (Abcam), cell permeabilization was done using 0.5% Triton X-100 (Sigma-Aldrich) in 2% FBS-PBS at +4°C for 30 min. Data was captured with a BD FACSCalibur and analyzed with FCS Express 6 Flow Research Edition software (*De Novo* Software).

The statistical analysis of the data was done using GraphPad Prism software (version 5.04, GraphPad Software Inc., CA, USA). One-way ANOVA tests were done for analyzing the group differences for both expression of endothelial markers between the treatments and as a function of time. If differences were statistically significant with more than 95% confidence, paired differences between the groups were tested with Tukey’s multiple comparison post-tests.

### Functional Characterization of Differentiated ECs

Tube forming capacity of the differentiated ECs at day 14–15 was tested on 10 mg/ml Matrigel Basement Membrane Matrix (BD Biosciences) according to manufacturer’s instructions. Briefly, 300 µl of Matrigel was plated per well of a 24-well culture plate kept on ice. Plates were incubated at +37°C for 50 min and 300 µl of cell suspension with 1.2 × 10^5^ cells in the EC differentiation medium with appropriate supplements were seeded per well. 50 µM L-sulforaphane (Sigma-Aldrich) was used to inhibit specific EC tube formation (41). Pictures of forming tubes were taken by a continuous live cell imaging system, Cell-IQ Analyzer (Chip-Man Technologies) supplied with a 10× objective 12–15 h after cell seeding.

Uptake of acetylated LDL (Ac-LDL) by differentiated ECs at day 14–15 was confirmed by Alexa Fluor^®^ 488 labeled Ac-LDL (L23380, Molecular Probes) at the final concentration of 1 µg/ml. Fibroblasts (ATCC, CRL-2429) cultured in endothelial cell growth medium (Thermo Scientific) on fibronectin-gelatin coating (10 µg/ml, 0.05%; Sigma-Aldrich) were used as negative controls. Cells were incubated with Ac-LDL for 3 h prior to fixing in 1% PFA - 2% FBS in PBS. Flow cytometric analyses were performed using BD FACSCalibur and FCS Express 6 Flow Research Edition software.

### RNA-Seq Libraries

RNA sequencing was performed to compare transcriptional profiles of different EC differentiation groups and hiPSCs. RNA was extracted from cells with RNeasy Mini Kit (Qiagen) and depleted from rRNAs using Ribo-Zero Gold Kit (Illumina). RNA was fragmented using TURBO DNase and RNA fragmentation reagents (Life Technologies) and purified using P-30 columns (Bio-Rad, Hercules, CA, USA). Fragmented RNA was dephosphorylated with polynucleotide kinase (New England Biolabs, Ipswich, MA, USA) followed by heat-inactivation and purification using RNA Clean & Concentrator™−5 kit (Zymo Research Corporation, Irvine, CA, USA). Poly(A)-tailing and reverse transcription were performed as previously described (42). Libraries were amplified using 11 cycles, size-selected (200–350 bp) in 10% TBE gels (Life Technologies) and sequenced using Illumina HiSeq 2000 for 50 cycles according to the manufacturer's instructions.

### RNA-Seq Data Analysis

RNA-Seq results were trimmed to remove 3′ A-stretches originating from the library preparation and poor quality reads were filtered out (minimum 97% of bp over quality cut off 10). Reads were aligned to the hg19 genome using Tophat allowing up to two mismatches and reporting only one alignment for each read. Data analysis was performed using HOMER (43) (http://homer.ucsd.edu/homer/) and the differential gene expression using edgeR (44). Thresholds of FDR < 0.05, reads per kb per million reads >0.5 and fold change >4 were used. Clustering results were generated by Cluster 3.0 (45) by normalizing and centering the gene expression tags to range from −1 to 1. The output from clustering was viewed using Java Treeview (46). The gene ontology (GO) analysis was performed using DAVID Bioinformatics Resources 6.8 (47) and gene lists for selected top functions were exported to generate clustered heatmaps. QIAGEN’s Ingenuity® Pathway Analysis (IPA®, QIAGEN Redwood City, www.qiagen.com/ingenuity) was used to perform Upstream Regulator Analysis with a focus on transcriptional regulators to identify which factors may be causing observed gene expression changes. Gene Expression Dynamics Inspector (GEDI) v2.1 (48) was used to generate self-organizing maps to visualize the expression profiles of different treatments and timepoints. Grid size 26 × 25, 1st phase training iteration 20 and 2nd phase training iteration 80, were used as settings to generate mosaic images. Principal component analysis (PCA) was performed using prcomp function in R environment and 3D plot was prepared using rgl R package.

### Data Access

RNA-Seq data from this study has been submitted to NCBI Gene Expression Omnibus under accession number GSE103945. 

## Results

### Optimization of EC Differentiation Method and Characterization of Cells

In this study, we systematically tested and compared the effects of the most potent signalling factors and small molecules reported to generate ECs from hiPSCs. Tested molecules included ROCK inhibitor indispensable for EC differentiation (25), TGFβ inhibitor that maintains vascular identity after the EC specification and support endothelial cell expansion (24, 49), cAMP analog 8-Br-cAMP that promotes differentiation of ECs (23, 31), and BMP-4 that contributes to mesodermal commitment (30). These molecules were used in seven different combinations (Table 1) added to the serum-free endothelial basal medium. In addition, well-known and essential growth factors for EC differentiation and proliferation, VEGF-A and FGF-2, were always included in EC culture media (50–52).

**Figure 1:**
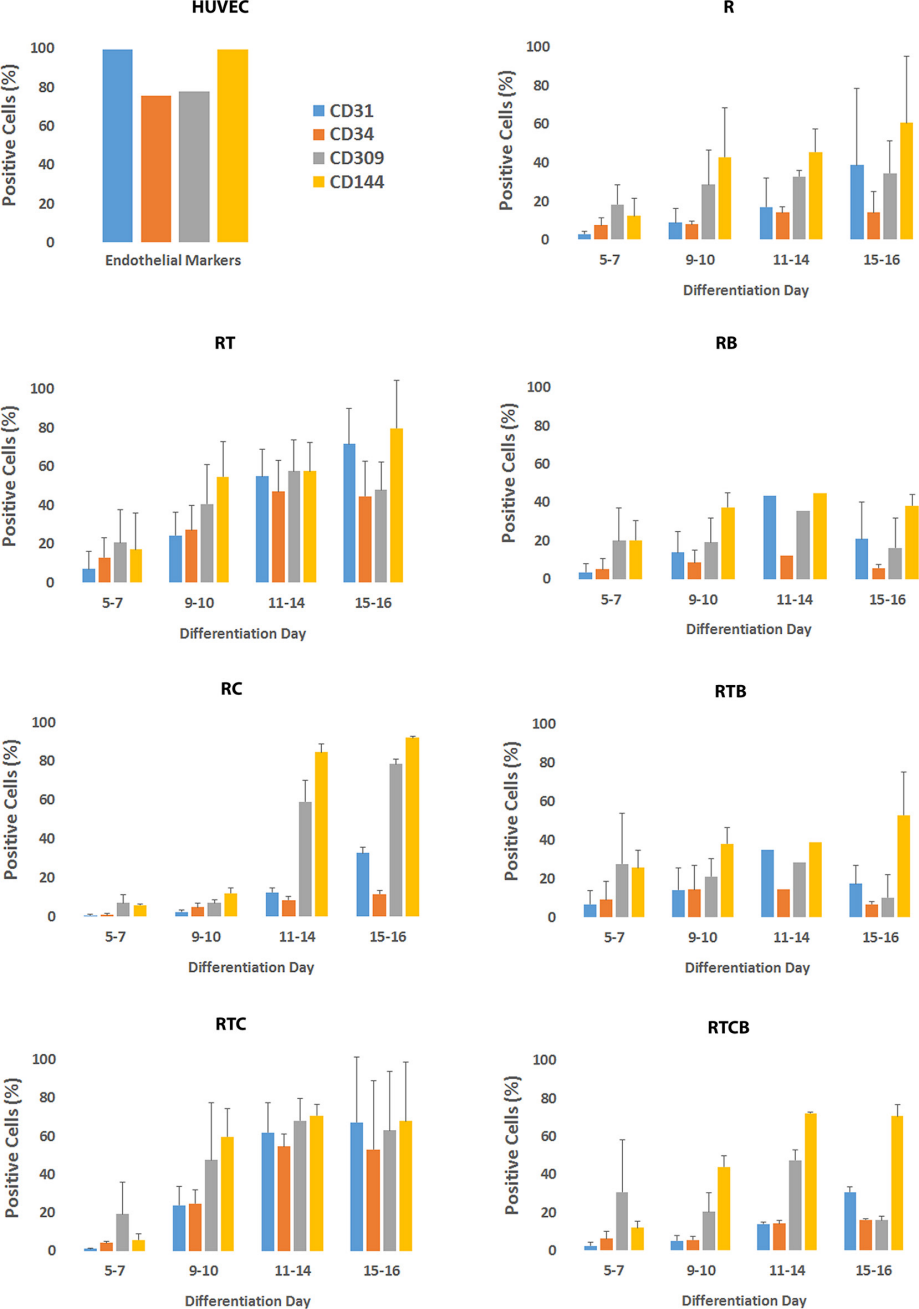
Phenotypic marker analysis of differentiating ECs. Bar charts represent the percentage of positive cells and SD based on flow cytometric analyses of CD31, CD34, CD309 and CD144 markers. Staining protocol was performed approximately the same days during differentiation depending on the proliferation of cells. Number of experiments done differed between treatment groups (R, *n* = 4; RT, *n* = 7; RB, *n* = 5; RC, *n* = 6; RTB, *n* = 5; RTC, *n* = 4; RTCB, *n* = 4).

**Figure 2:**
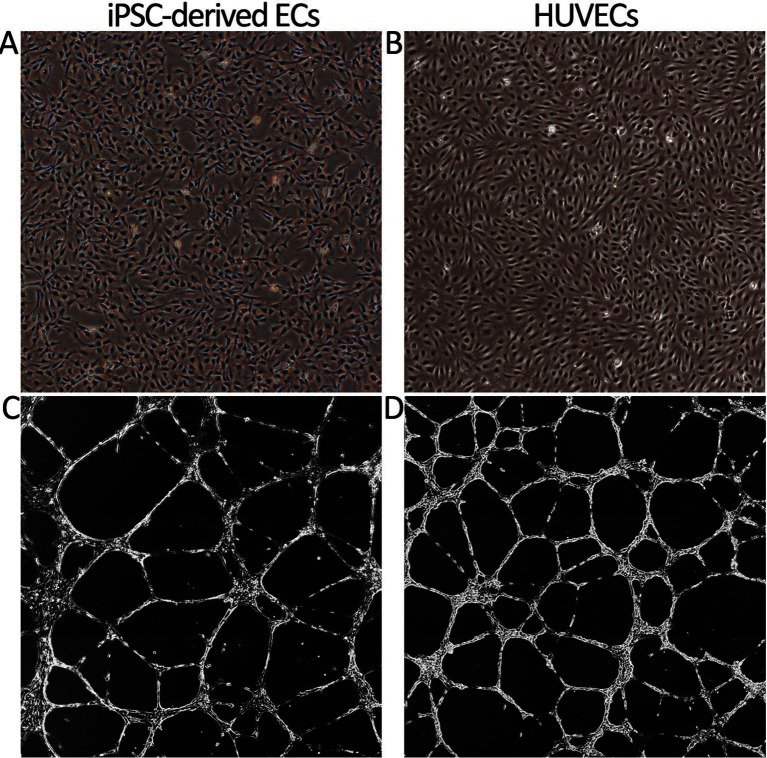
Cell morphology and tube formation on Matrigel of iPSC-derived ECs and primary HUVECs. **(A)** The morphology of iPSC-derived ECs at day 16 (group RTC). Prolonged culturing resulted in a change of morphology towards spindle shape resembling fibroblasts (data not shown). **(B)** HUVEC cobble stone morphology. Images are taken with original magnification of 40×. **(C)** IPSC-derived ECs cultured on Matrigel form tube networks. The picture is a presentative example of tube forming for different EC differentiation groups (group RT). **(D)** Tube formation of HUVECs. Stitched images of tube formation are created by Cell-IQ Analyzer using 10× objective.

The optimal time necessary for EC differentiation was determined by culturing hiPSCs in seven different conditions up to 36 days. At the end of the assay, endothelial markers were expressed only in low number of cells (Figure S1). This allowed us to conclude that differentiation of hiPSCs into mature ECs takes approximately 15 days, which is well in accordance with previous literature (53). As seen in Figure 1, a similar trend in cell surface marker expression was seen in all EC treatment groups where the number of cells expressing generally used EC markers, CD31 (PECAM1), CD34, CD309 (vascular endothelial growth factor receptor 2, VEGFR-2, KDR) and CD144 (VE-cadherin, CDH5), was increasing during the first two weeks. Treatments groups did not differ statistically at the end of differentiation (day 15–16) based on all four cell surface markers analyzed although there were statistical differences between single markers. Differentiating ECs also showed similar morphology across different cell culture groups at day 15 resembling the appearance of HUVECs (representative images in Figure 2A–B).

Lack of the ROCK inhibitor in the cell differentiation media resulted in a poor attachment of cells onto fibronectin-gelatin coating. Indeed, ROCK suppression has been shown to be indispensable to EC proliferation and differentiation from PSCs (25), and therefore it was included in all cell culture conditions. Consequently, the treatment group containing only the ROCK inhibitor (R) served as a reference group in the following studies. When the TGFβ inhibitor was included with the ROCK inhibitor from the beginning of the differentiation (RT), EC differentiation was promoted and more than 50% of the cells expressed CD31 and CD144 markers at day 15 (Figure 1). Addition of 8-Br-cAMP (RTC) did not have a major effect on the marker expression in comparison with the RT group. When comparing group RC to the reference group R, especially marker CD309 and CD144 expressions were higher. In contrast, adding BMP-4 at day 1 resulted in the EC marker suppression (RB, RTB and RTCB groups). HUVECs were used as positive controls and almost 100% of the cells were positive for CD31 and CD144. In comparison, the number of HUVECs positive for CD34 and CD309 were approximately 70%.

**Figure 3:**
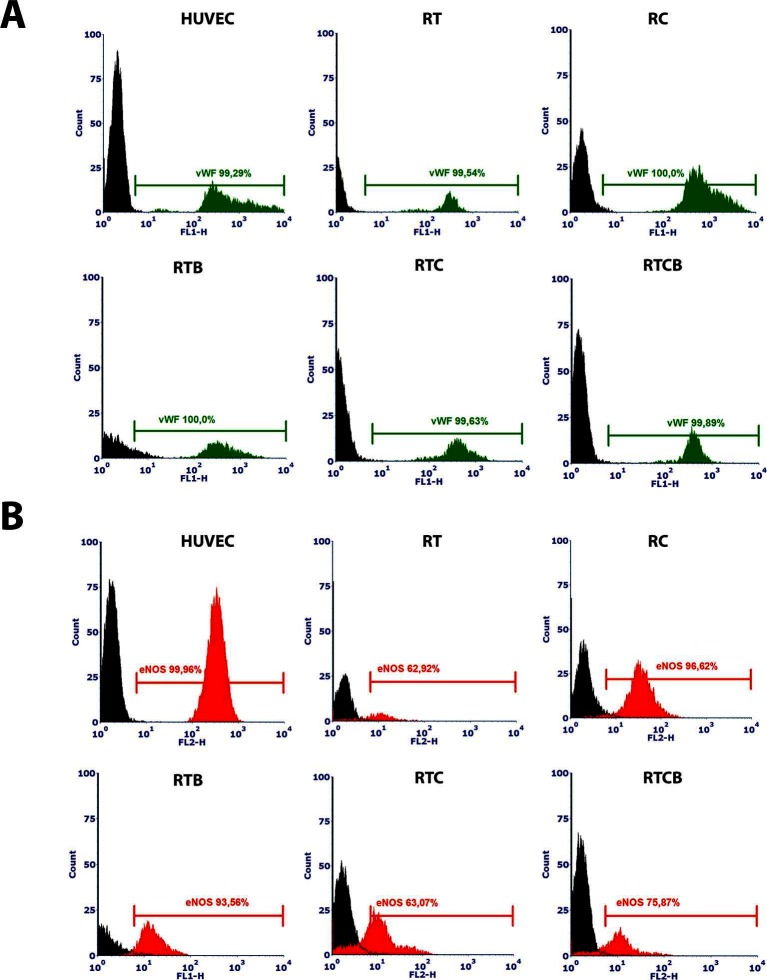
Intracellular vWF **(3A)** and eNOS **(3B)** phenotype markers in different treatment groups at day 16. HUVECs were used as a positive control. Differentiated ECs showed a similar expression pattern of the markers in all tested cell culture groups, but the highest percentage of positively stained cells for eNOS was in RC group. The results of the stainings were similar between different experiments and they showed high reproducibility (data not shown). The numbers of experiments done: RT, *n* = 1; RC, *n* = 4; RTB, *n* = 3; RTC, *n* = 1; RTCB, *n* = 3.

In addition, expression of intracellular EC markers vWF and eNOS was studied. As shown in Figure 3A, vWF was highly and uniformly expressed in RT, RC, RTB, RTC and RTCB groups and resembled the expression profile seen in HUVECs. eNOS was also expressed in all treatment groups but the number of positive cells was lower than in HUVECs (Figure 3B). The highest percentage of positive cells for eNOS (approximately 96%) was in RC group.

### Functional Assays of Differentiated ECs

**Figure 4:**
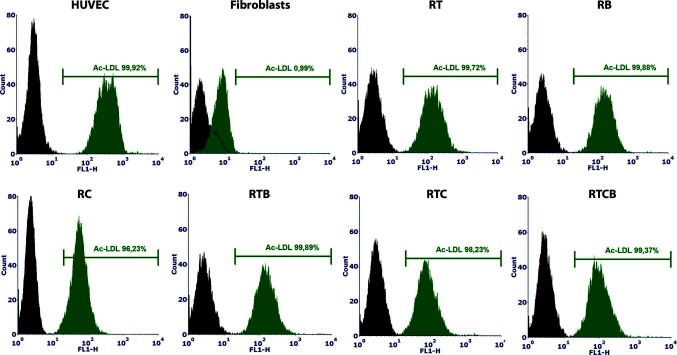
Ac-LDL uptake assay analyzed by flow cytometry using Alexa Fluor^®^ 488 labeled Ac-LDL. HUVECs and fibroblasts were used as positive and negative controls, respectively. HUVECs were 100% positive for Ac-LDL and among fibroblasts, only 1% were positive. Differentiated ECs (*n* = 1) showed similar percentages of internalized Ac-LDL (97–100%) to HUVECs at day 15.

Next, we studied the functional characteristics of ECs originating from different treatment groups. First, tube formation assay was employed to demonstrate the angiogenic activity of ECs. Our analysis demonstrated that a similar tube formation potential on Matrigel was evident in all tested conditions compared to mature ECs (HUVECs) (Figure 2C–D). The tube formation was inhibited using 50 µM sulforaphane (data not shown). Secondly, we studied the capacity of the differentiated ECs to take up Ac-LDL. In all tested EC culture groups, cells were able to internalize Ac-LDL (Figure 4).

### RNA Sequencing

**Figure 5:**
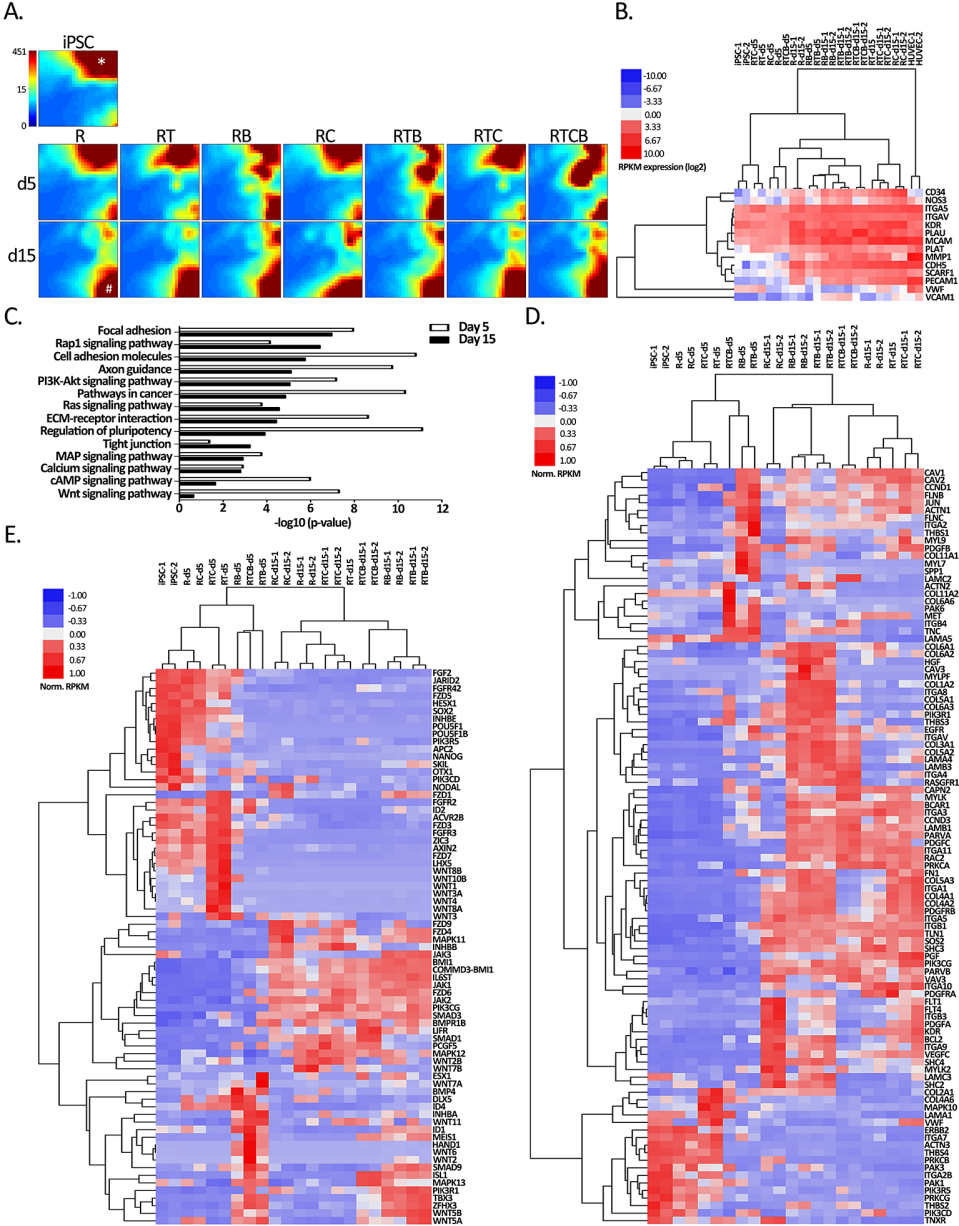
Gene expression analyses of hiPSC compared to all EC treatment groups. **(A)** GEDI analysis of the genes differentially regulated (hiPSC vs treatment FC >4) at least in one differentiation regimen at day 15 (*n* = 8535 RefSeq genes). Each tile represents a cluster of genes with similar expression profiles across the samples. The color indicates the expression strength of a gene cluster (blue, low expression; red, high expression). Star shows a gene cluster highly expressed in iPSCs that correspond to pluripotency genes (data not shown) and hash mark shows a gene cluster induced during differentiation at day 15 related to focal adhesion (data not shown). **(B)** Heatmap of EC markers used in flow cytometric analyses (*CD34*, *NOS3*, *CDH5*, *KDR*, *PECAM1* and *VWF*) and genes related to EC functional assays. Spearman’s rank correlation was used for sample clustering. **(C)** KEGG pathways (DAVID) of differentially expressed genes in at least one differentiation condition at day 5 (*n* = 6924) or day 15 (*n* = 8535). **(D–E)** Hierarchical clustering of genes associated with **(D)** focal adhesion and **(E)** regulation of pluripotency functions from B based on average correlation.

The gene expression profiles of differentiating ECs were studied in more detail using RNA-Seq. For transcriptome analysis, cells were treated as described above and RNA was isolated at days 5 and 15 upon differentiation. Single replicates of day 5 were used to visualize the intermediate mesodermal stage, whereas two replicates were used for more throughout analysis of the end point of differentiation at day 15 and for the control hiPSC samples. The two timepoints were clearly separated by principal component analysis (PCA) (Figure S2A), with iPSC cells located next to day 5 samples and day 15 farther apart. Along the PC1 axis, which contributes most to the variance in the dataset (36% *vs* 17% for PC2), the day 15 samples situated closest to HAEC and HUVEC controls supporting successful reprogramming. Also the two replicates exhibited high reproducibility by PCA (Figure S2B). The expression of EC markers studied by flow cytometry analyses was well in concordance with the sequencing results, suggesting that changes on the transcript levels were translated to a similar profile on the protein level (Figure 5B and Table S1). We also studied the expression of endothelial LDL scavenger receptor SCARF1 responsible for the uptake of Ac-LDL (53–56) and adhesion proteins and enzymes involved in ECM processing related to tube forming ability. At mesodermal phase at day 5, differentiating cells had lower expression of EC-related genes except vWF. Groups RB, RTB and RTCB clustered together at day 15 suggesting a significant connective role of BMP-4 for these groups (Table S1). In these groups, the expression of EC-related genes was the lowest which reflects the results seen visually in flow cytometric analyses even though no statistically significant differences between treatment groups was found (Figure 1). Among them, group RTCB had the lowest expression of EC genes while the expression of *MMP1* and *PLAU* was high in this group. Interestingly, at a differentiation day 15, treatment group RC had more upregulated EC genes (*CD34*, *NOS3*,* KDR*) than the other treatment groups. RC group did not yet express EC surface markers on day 10 as evidenced by FACS (Figure 1) but the expression of EC markers took off by day 11. This suggests that the differentiation process is slower although still successful in this group.

First, we analyzed how the landscape of transcripts was changed in the differentiation groups compared to the hiPSC reference using the Gene Expression Dynamics Inspector (GEDI) analysis (48). Altogether 8535 RefSeq transcripts, corresponding to 4136 different genes, were found differentially regulated in at least one differentiation regimen at day 15 (FDR <0.05, FC > 4, RPKM > 0.5; Table S1). Majority (3690/4136) corresponded to protein-coding accessions (NM_), whereas the remaining 11% represented non-coding RNAs (NR_). GEDI genomic landscape maps demonstrated that the gene expression patterns of hiPSCs and differentiation groups at day 5 were more similar to each other than the day 15 groups (Figure 5A). Additionally, resulting GEDI representations also revealed that a distinctive EC-specific gene expression profile began to emerge in RB, RTB and RTCB groups already at day 5 (Figure 5A). However, by day 15 a more prominent shift in gene expression pattern was seen with shutting down of genes related to regulation of pluripotency and activation of genes related to focal adhesion in all groups (Figure 5A, star/hash, respectively). In line with this, the GO analysis revealed that pathways and signaling related to pluripotency and cellular adhesion, including focal adhesion, cell adhesion molecules, ECM-receptor interactions and tight junctions, were significantly enriched among the differentially regulated genes (Figure 5C). Notably, the pluripotency genes were more highly enriched at day 5 whereas at mature cell level the top GO term was associated with focal adhesion.

In Figure 5D–E, hierarchical clustering of genes related to focal adhesion and pluripotency demonstrated faster gene expression changes for RB, RTB and RTBC groups at day 5 and distinct upregulation of mesodermal genes *INHBA*, *BMP4*, *MEIS1*, *HAND1* and *TBX3*. In contrast, a higher similarity of all differentiation groups was seen especially in pluripotency related gene expression pattern at day 15. However, the RC group demonstrated the highest expression of VEGF-family members, including *FLT1*, *FLT4*, *KDR* and *VEGFC* in line with the role of cAMP signaling promoting the survival of VEGFR-2 positive cells (31) and TGFβ inhibition in maintaining vascular identity after mesodermal fate (24). In addition, a larger group of adhesion molecules, including integrins and collagens, were activated with the three conditions including BMP-4 treatment at day 15 (Figure 5D). Moreover, TFs, such as mesodermal related *MEIS1*, *HAND1* and *TBX3* (57, 58) (http://pathcards.genecards.org/card/mesodermal_commitment_pathway) together with *SMAD9*, *ISL1* and *ZFHX3*, were highly induced by the BMP-4 groups suggesting distinct differentiation signatures. This suggests that a distinct set of TFs could be induced by different cell culture conditions.

**Figure 6:**
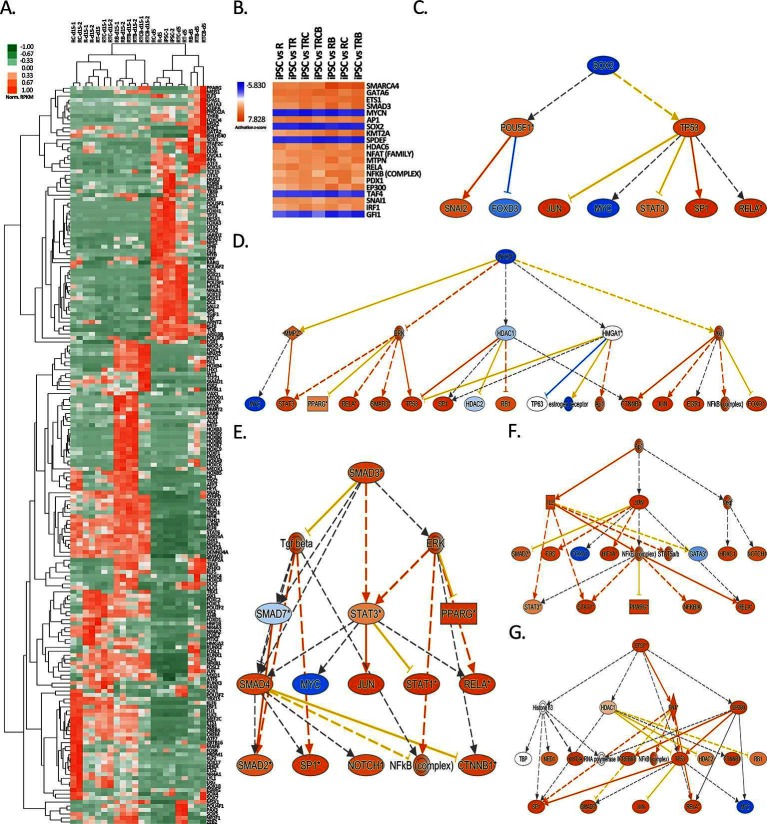
Identification of top upstream regulators responsible for gene expression changes during EC differentiation. **(A)** Heatmap of normalized RPKM values (−1 to 1) of the top 186 transcription factors differentially regulated over 100-fold in response to differentiation treatments. Hierarchical clustering (average linkage) was performed for genes and samples. **(B)** IPA analysis of upstream transcription regulators for hiPSC *versus* EC treatment groups shown for the 20 top upstream regulators with the highest z-scores. **(C-G)** Mechanistic networks generated by IPA for selected transcription factors from B. Blue color stands for predicted inhibition and orange for predicted activation. The tones of color indicate confidence level (light = low confidence; dark = high confidence). Transcription factors **(C)***SOX2* and **(D)***MYCN* were predicted to be inhibited and **(E)***SMAD3*, **(F)***AP1* and **(G)*** ETS1* activated.

It has been suggested that there are master regulators that control lineage-specific gene expression and such TFs would be highly expressed in differentiated cell types (59–61). Therefore, we studied the expression of differentially regulated TFs between the hiPSC and various EC differentiation conditions. Our analysis identified 186 TFs, which clearly clustered the samples based on the differentiation stage (Figure 6A). For example, pluripotency related TFs *POU5F1* (OCT-4), *SOX2*, *JARID2* and *GLI1* were highly repressed in all treatment groups except R and RC at day 5. The clustering of R/RC together with hiPSC samples at day 5 also suggests that 8-Br-cAMP does do not promote early cell differentiation. On the other hand, RT/RTC groups already exhibited repression of pluripotency genes, suggesting that early TGFβ inhibition promotes differentiation at day 5. Notably, the most striking difference was seen for the RB, RTB and RTBC groups that clustered away from the other treatment groups at day 5. This was most attributable to the early repression of *MYCN*, *SOX2*, *SOX21*, *SOX11*, *SOX13* and *POU3F1* genes, which was evident in the other treatment groups only at day 15. Additionally, these groups also exhibited a high expression level of mesodermal TFs *GATA2* and *GATA3* at day 5. This likely reflects the ability of exogenous BMP-4 to promote differentiation of pluripotent cells toward mesodermal cells (33).

As a demonstration of the success of differentiation, the major EC-specific TFs, such as *ETS1*, *ETS2*, *JUNB*, *ERG*, *SOX7*, *SOX17*, *SOX18*, *HHEX*, *ELK3*, *KLF6*, *MEF2C* and *FLI1* (62–68) were highly induced in the majority of differentiation groups at day 15 (Figure 6A). The highest expression of these crucial EC-related TFs was in RC group and the lowest in RTCB group. This suggests that RC, with the highest expression of EC markers (Figure 5B) and EC-specific TFs (Figure 6A) likely represents the most EC-like phenotype. This result was further confirmed by studying the expression of 50 most highly expressed TFs in HUVECs and HAECs, where RC group clustered closest to these mature EC types (Figure S3).

To study how changes in the TF expression could explain the global changes in gene expression patterns, we further searched for other upstream transcriptional regulators using Ingenuity Pathway Analysis (IPA). In line with TF expression, our analysis identified *MYCN* and *SOX2* as the top transcriptional regulators inhibited during differentiation, whereas *SMAD3*, *ETS1* and *AP1 *signaling was activated (Figure 6B–G). Although not directly linked with the induction of the respective TF, also *SMARCA4*, *GATA6* and *KMT2A* were identified as possible activated upstream regulators (Figure S4). These TF networks could thus explain gene expression changes during EC differentiation. Interestingly, a similar pattern of activation was also highly associated with a chemical compound tretinoin (Figure S5).

## Discussion

In this study, we focused on systematically testing and comparing the efficacy of the most potent signalling factors and small molecules used to produce ECs from hiPSC. Stem cells are a renewable, limitless source of differentiating ECs. They are also less immunogenic due to the use of autologous patient-specific cells, making them an attractive therapeutic tool (21, 69). However, iPSCs have their own disadvantages related to the risk of teratomas because of the pluripotent nature of the cells (69). There are also concerns related to incorporation of the iPSC generating viral vectors with the host genome and continuing transcription of transgenes in iPSC-derived, differentiated cells (21). Concisely, it is essential to test all possible EC types in a clinical setting to evaluate which cells have the most potential for therapeutic applications. We present here the first systematic characterization of the existing EC differentiation protocols by testing the effect of relevant signaling factors and small molecules, and evaluating the subsequent efficacy of EC differentiation from hiPSC. We used a simple, 2D monolayer cell culture with serum-free, well-defined cell culture medium to rigorously control factors affecting EC differentiation. EC differentiation took approximately 15 days and was confirmed by EC marker expression with flow cytometry analysis. EC maturation was further confirmed by functional EC assays, such as tube formation and Ac-LDL uptake, which demonstrated similar functional characteristics of differentiated ECs compared to HUVECs.

RNA-Seq was used to provide a genome-wide view of the gene expression changes during the seven different treatment protocols. To our surprise, all treatment groups exhibited many similarities at day 15, exemplified by silencing of genes related to regulation of pluripotency and upregulation of genes related to focal adhesion. The high similarity of groups also indicates that one factor in common to all, the ROCK inhibitor, a major downstream effector protein RhoA, is indispensable for EC differentiation (25). RhoA controls diverse array of cellular processes such as cytoskeletal dynamics, cell polarity, membrane transport and gene expression (70). ROCK inhibitors controls the expression of adhesion molecules and accordingly, ROCKs have been shown to affect cell–cell adhesion of ECs, and to regulate the integrity of cellular junctions (71). The effects of ROCKs are also linked to cAMP signalling via exchange protein directly activated by cAMP (Epac) signalling (72). Epacs are guanine nucleotide exchange factors (GEFs) that bind to cAMP. This pathway enhances EC barrier function by influencing EC junctional protein and actin cytoskeleton organisation. It downregulates RhoA activation and stress fiber formation (73). Recently, ROCK inhibition has been associated with the inhibition of endothelial-to-mesenchymal transition (74). The interaction between ECs and extracellular matrix influences key signaling events involved in EC migration, invasion, proliferation, and survival that are indispensable for angiogenesis (13). Our results clearly demonstrate that the majority of the gene expression changes occuring during EC differentiation are related to the regulation of genes and pathways associated with cellular adhesion and ECM-receptor interaction supporting their central role in inducing and maintaining EC function.

As a further proof of differentiation, major EC-specific TFs, such as *ETS1*, *ETS2*, *JUNB*, *ERG*, *SOX7*, *SOX17*, *SOX18*, *HHEX*, *ELK3*, *KLF6*, *MEF2C* and *FLI1* (62–68) were highly expressed in the majority of differentiation groups at day 15. Ingenuity Pathway Analysis was used to reveal upstream transcriptional regulators that could explain changes in the gene expression patterns during our differentiation procedure from hiPSCs to mature ECs. We identified *MYCN* and *SOX2* as the top transcriptional regulators that were inhibited, while *SMAD3* (75), *ETS1* (62) and *AP1* (76) signalling was stimulated. This information could provide a future means for the generation of more efficient EC differentiation protocols through the modulation of TF expression or small molecule drugs targeting TF function (63, 66, 67, 77). Interestingly, a chemical compound tretinoin was shown to induce highly similar gene regulation networks suggesting that it could be used to further enhance EC differentiation. Supporting this finding, a natural retinoid all-trans retinoic acid has been shown to promote angiogenesis by stimulating EC proliferation and enhancing endogenous VEGF signaling (78–80).

Our analysis of TF expression also revealed that the treatment group RC had the highest expression of EC-specific TFs together with the strongest expression of EC marker genes and VEGF family members. This suggests that, in addition to the ROCK inhibition, the supplementation of a cAMP analog 8-Br-cAMP in the beginning of differentiation could promote the most efficient differentiation. CAMP-signalling has been shown to be associated with Notch and protein kinase A (PKA)/Epac pathways. Activating these signalling pathways promotes differentiation and proliferation of ECs and survival of VEGFR-2 positive cells (23, 31), enhances EC barrier function (73), and activates eNOS which is responsible for, for example, EC-mediated vasorelaxation (81). Interestingly, the expression of eNOS on protein and RNA level was highest in RC and RTB groups and lowest in RT, RTC and RTCB groups. These differences could be due to complex impacts of TGFβ and cAMP signalling on eNOS expression and activity (81, 82). It has been shown that TGFβ increases the expression of eNOS acting via Smad2 signalling (82) and inhibiting this TGFβ pathway might lower the eNOS expression shown in our results. On the other hand, cAMP signaling has been shown to enhance eNOS activity through PKA/Epac signalling that further activates PI3K/Akt pathway (81).

Our results for the RC group also demonstrate that the effects of cAMP signalling take at least two weeks to occur, since only little gene expression changes were seen at day 5 and expression of EC markers analyzed by flow cytometry were not yet evident at day 10. An explanation for the slow differentiation can be explained by the fact that the RC group received the TGFβ inhibitor at day 4, in contrast to the RTC group which was cultivated in the presence of this inhibitor throughout the time course. TGFβ signalling has many roles including cell differentiation, migration and maintaining pluripotency of stem cells. The role of TGFβ signalling in maintaining pluripotency of PSCs is controversial, some studies supporting its role in pluripotency maintenance and others confronting this assumption (83–85). The TGFβ inhibitor SB431542 used in these experiments inhibits signalling mediated by ALK-4, ALK-5 and ALK-7 receptor while leaving ALK1 signalling unaffected (86). Signalling activated by ALK-5 inhibits EC proliferation, tube formation, and migration (87). In line with this, the TGFβ inhibitor has shown to enhance EC differentiation from ESCs and EC growth (35, 50), and it is needed in the maintenance of the vascular identity after EC specification (24). Additionally, it has been shown that TGFβ inhibition functionalizes VEGFR-2 signalling (88). Altogether, TGFβ signalling has multiple roles in several cell types and it it essential to balance between those counteracting functions in EC differentiation protocols. Our results provide evidence that TGFβ inhibitor is most useful in EC differentiation when added after mesodermal induction to promote subsequent EC spesification.

Interestingly, our results indicated high expression of mesodermal genes including *INHBA*, *BMP4*, *GATA2*, *GATA3*, *MEIS1*, *HAND1*, *KLF5* and *TBX3* (57) in the treatment groups receiving BMP-4 at day 5. This is in line with findings that exogenous BMP-4 promotes early mesodermal differentiation (30, 33, 89, 90). The BMP-4 activation, however, did not help in gaining a mature EC phenotype. However, groups RB, RTB and RTCB clustered together at day 15 and exhibited distinct gene signatures exemplified by the induction of a large group of integrins and collagens. This suggest a significant connective role of BMP-4 in these groups although it does not specifically promote mature endothelial differentiation.

In conclusion, we used a simple, serum-free 2D monolayer cell culture method to guide hiPSC into ECs, omitting complicated manufacturing procedures. Using human iPSC as source material and ECs derived from them enables autologous or allogeneic cell preparations to be tested in studies aiming at regenerative vascularization. EC differentiation took approximately 15 days. Differentiated cells showed a typical pattern of EC surface antigens and functional properties, such as tube formation and Ac-LDL uptake. Transcriptomic profiling demonstrated that although the treatment groups were highly similar at day 15, the most potent factors inducing EC phenotype were the cAMP analog 8-Br-cAMP employed at the beginning of EC differentiation and the TGFβ inhibitor SB431542 added after the mesodermal differentiation at day 4. Additionally, the ROCK inhibitor Y-27632 is highly beneficial to EC differentiation and it should be included in the EC culture media. It was also shown that exogenous BMP-4 supplemented from day 1 to day 4 activates early mesodermal differentiation but gives no advantage later in the differentiation process when cells are gaining mature EC phenotype. In summary, this optimized cell culture method provides an improved basis for an efficient EC production from hiPSCs, and offers invaluable information about the transcriptional changes occurring during the EC differentiation that could be employed in the generation of ECs for regenerative therapy applications.

## Ethics Statement

HUVECs were isolated from umbilical cords obtained from the maternity ward of the Kuopio University Hospital under the approval of the Hospital Ethics Committee (permit 341/2015). The samples were anonymous and cannot be traced back to the donor.

## Author Contributions

HB and JKK designed the study. HB performed experiments and analyzed the data. JKK participated in the research and data analysis and revised the manuscript. TK coordinated research and revised the manuscript. KP provided stem cells, coordinated stem cell work and revised the manuscript. PIM performed flow cytometry analyses and prepared pictures related to them. HN contributed to the RNA-Seq data analyses. JO performed statistical analyses. GW participated in manuscript preparation and revision. JK provided stem cell facilities and enabled research using stem cells. MK coordinated research, analyzed and prepared figures of RNA-Seq data. HB and MK wrote the manuscript. SY-H contributed to the conception and design of the research, critically revised the manuscript for important intellectual content, and supervised the research.

## Conflict of Interest Statement

The authors declare that the research was conducted in the absence of any commercial or financial relationships that could be construed as a potential conflict of interest.

## Supplementary material

Figure S1 EC differentiation duration from iPSC was tested by monitoring of ECmarker expression up to 36 days. A presentative diagram of differentiation group RT showing howEC marker expression goes up until differentiation day 15–16 and down after that. ECs lose theirphenotype in a longer culturing period in all tested differentiation groups.

Figure S2 Principal component analysis (PCA) of log-transformed RPKM valuesfrom **(A)** all RNA-Seq samples (2D plot) and **(B)** all samples subjected to 15 day differentiationregimen (3D plot).

Figure S3 Heatmap of normalized RPKM values (−1 to 1) of the 50 most highly expressed transcription factors in HUVECs and HAECs. Clustering was performed for genes and samples using Spearman's rank correlation (complete linkage).

Figure S4 Mechanistic networks generated by IPA for transcription factors SMARCA4, GATA6 and KMT2A predicted to be activated from Figure 6B. Blue depicts predicted inhibition and orange activation. The tones of color indicate confidence level (light = low confidence; dark = high confidence).

Figure S5 Mechanistic networks generated by IPA for a chemical compoundtretinoin predicted to be activated. Blue depicts predicted inhibition and orange activation. The tonesof color indicate confidence level (light = low confidence; dark = high confidence).

Table S1 Gene expression analysis comparing hiPSCs to treatment groups on day 5 and day 15. Normalized gene expression values are provided with log2 FC and FDR values for each pairwise comparison.
