# Supplementary figures and images for: Temporal Dynamics of Gene Expression During Endothelial Cell Differentiation From Human iPS Cells: A Comparison Study of Signalling Factors and Small Molecules
Source: Front Cardiovasc Med. 2018 Mar 14;5:16. doi: 10.3389/fcvm.2018.00016 (PMC5861200; doi:10.3389/fcvm.2018.00016)

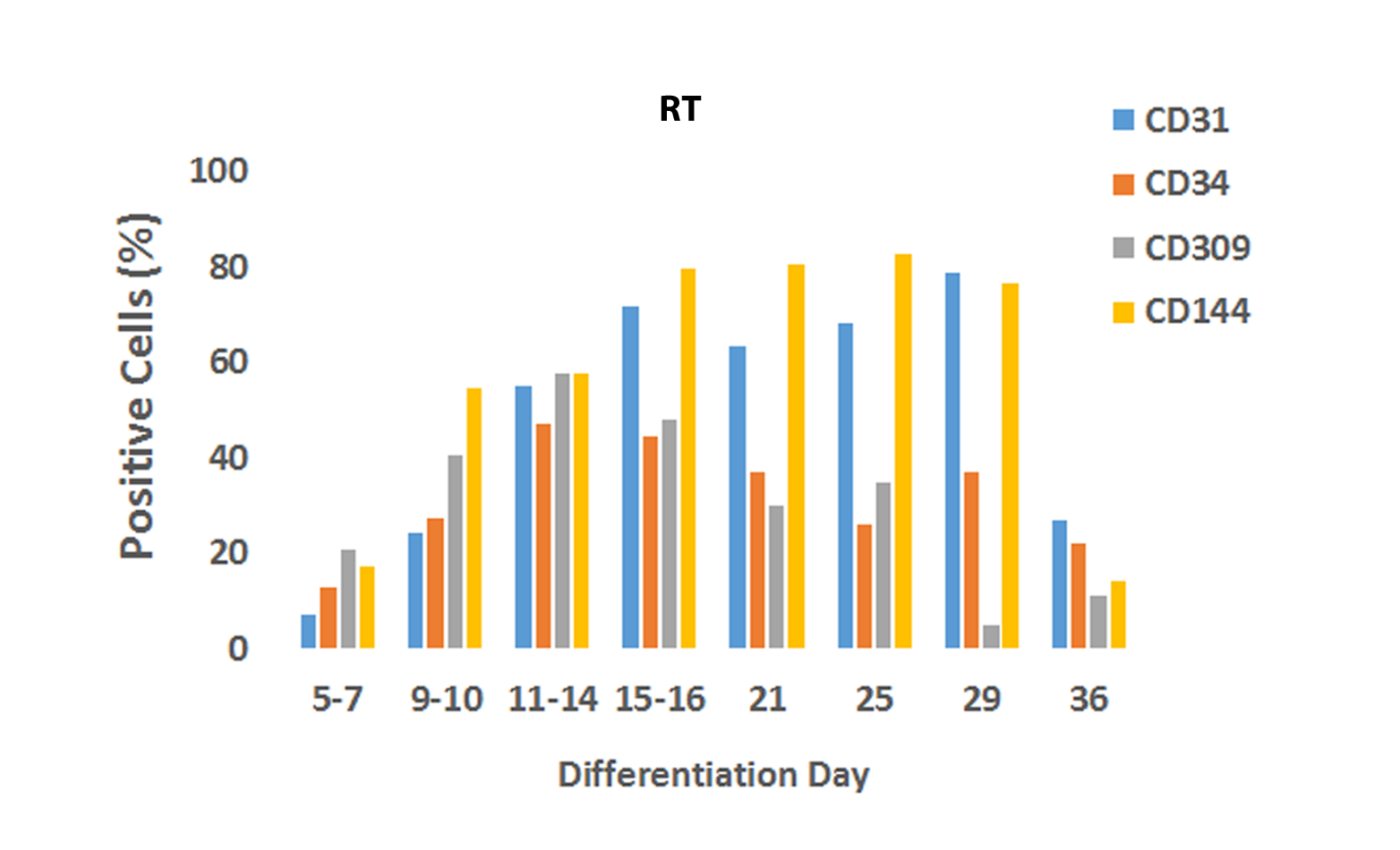

Supplement: Figure S1 — EC differentiation duration from iPSC was tested by monitoring of ECmarker expression up to 36 days. A presentative diagram of differentiation group RT showing howEC marker expression goes up until differentiation day 15–16 and down after that. ECs lose theirphenotype in a longer culturing period in all tested differentiation groups. [file Image1.tif]

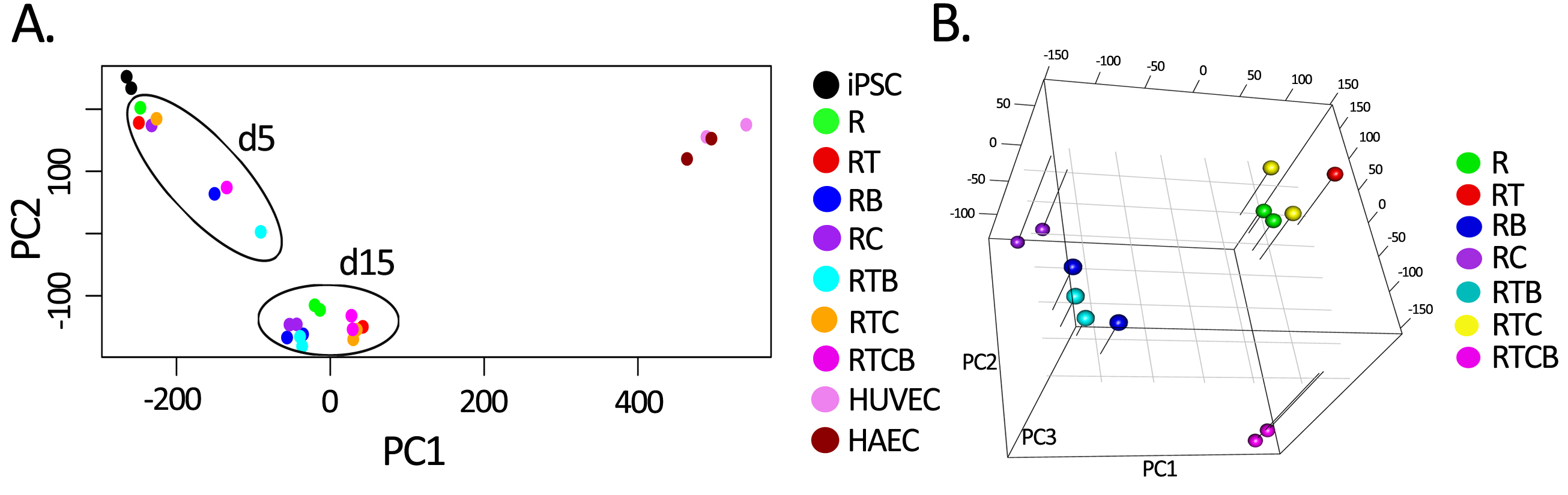

Supplement: Figure S2 — Principal component analysis (PCA) of log-transformed RPKM valuesfrom (A) all RNA-Seq samples (2D plot) and (B) all samples subjected to 15 day differentiationregimen (3D plot). [file Image2.jpeg]

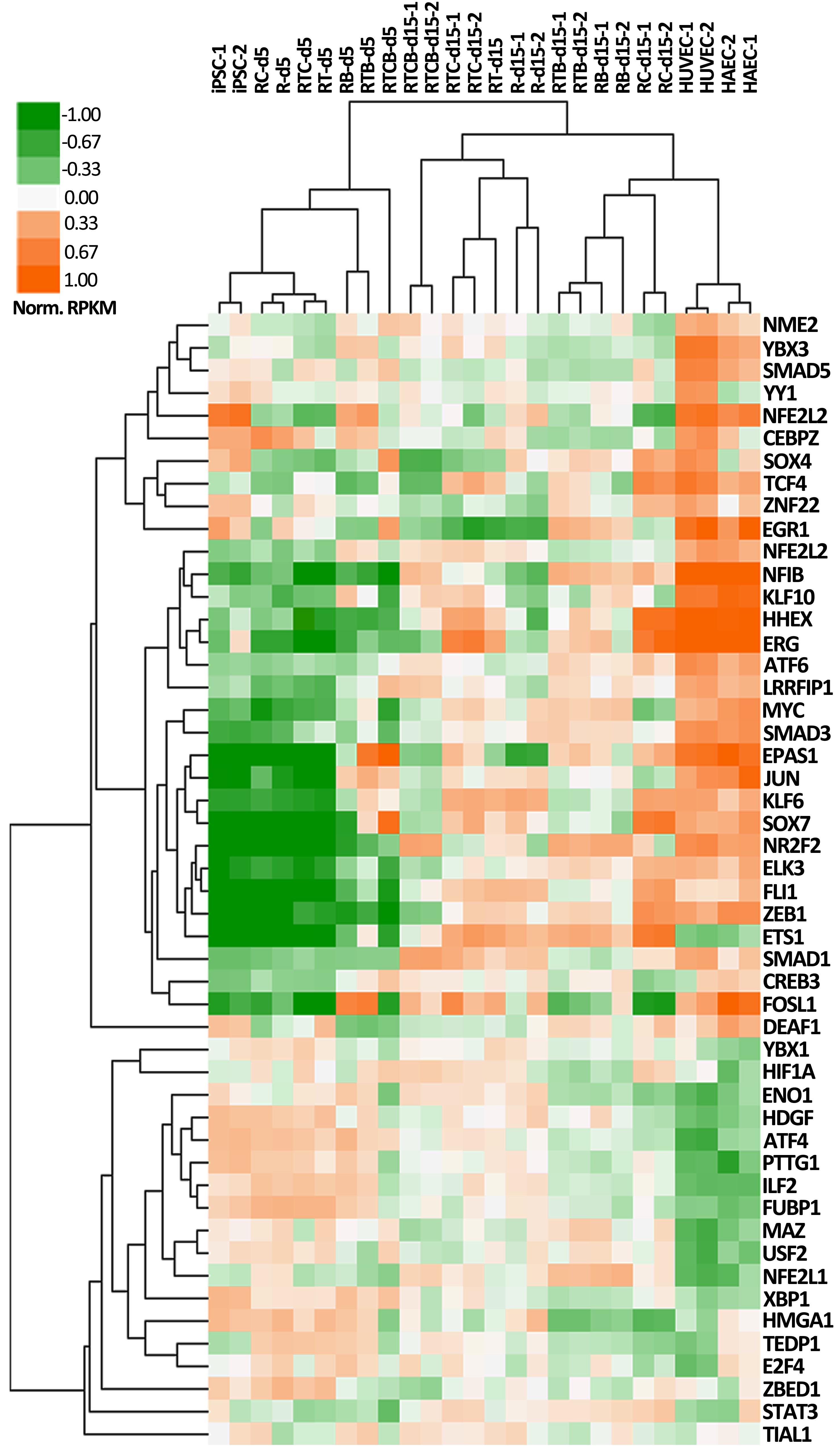

Supplement: Figure S3 — Heatmap of normalized RPKM values (−1 to 1) of the 50 most highly expressed transcription factors in HUVECs and HAECs. Clustering was performed for genes and samples using Spearman's rank correlation (complete linkage). [file Image3.jpeg]

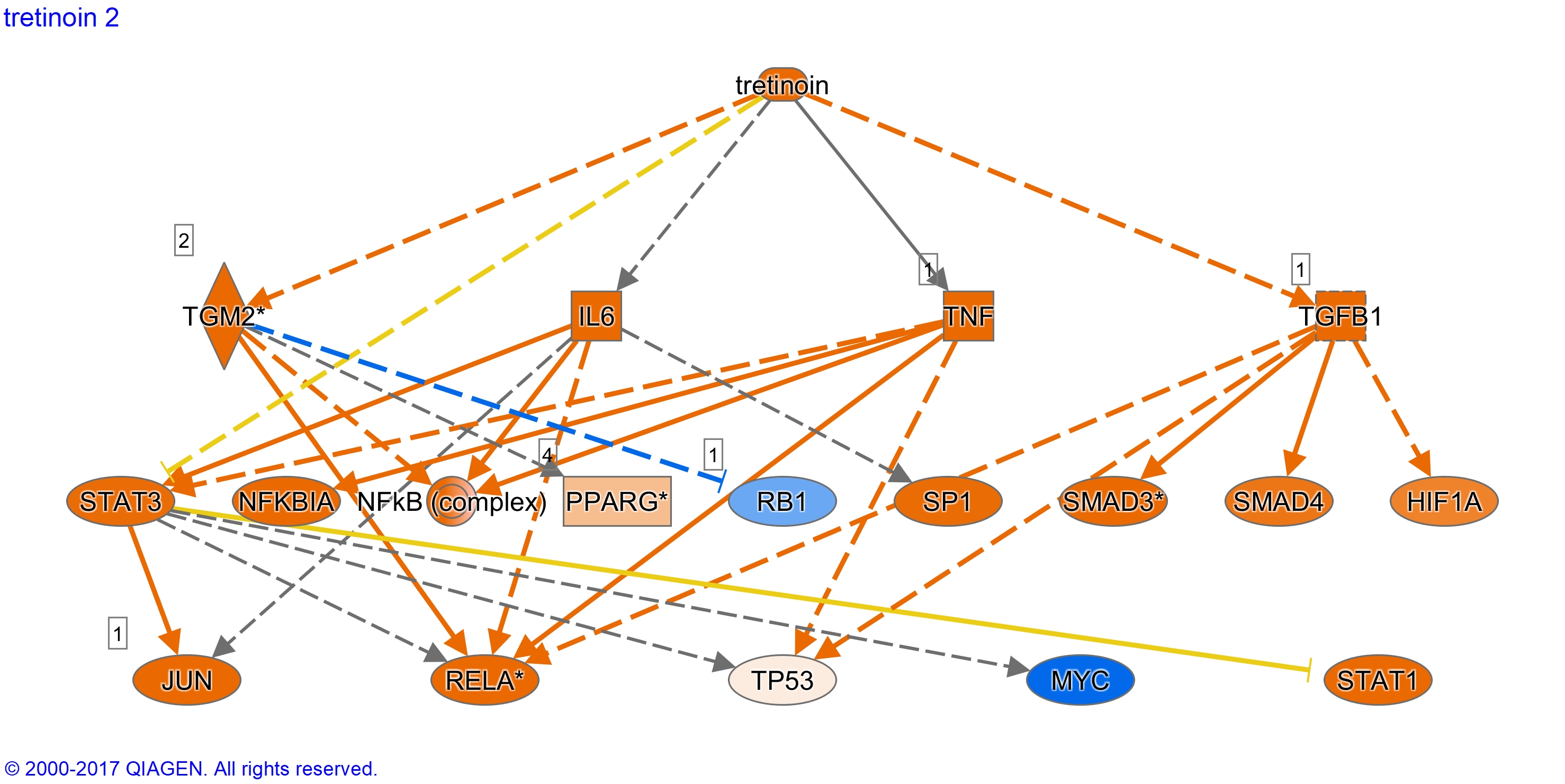

Supplement: Figure S5 — Mechanistic networks generated by IPA for a chemical compoundtretinoin predicted to be activated. Blue depicts predicted inhibition and orange activation. The tonesof color indicate confidence level (light = low confidence; dark = high confidence). [file Image5.jpeg]
